# The Role of Curcumin in Preventing Naturally Occurring Leiomyoma in the Galline Model

**DOI:** 10.3390/ph17121732

**Published:** 2024-12-21

**Authors:** Kazim Sahin, Cemal Orhan, Mehmet Tuzcu, Nurhan Sahin, Ibrahim H. Ozercan, Nashwa Kabil, Omer Kucuk, Bulent Ozpolat

**Affiliations:** 1 Department of Animal Nutrition, Faculty of Veterinary Medicine, Firat University, 23119 Elazig, Turkey; corhan@firat.edu.tr (C.O.); nsahin@firat.edu.tr (N.S.); 2 Department of Biology, Faculty of Science, Firat University, 23119 Elazig, Turkey; mtuzcu@firat.edu.tr; 3 Department of Pathology, Faculty of Medicine, Firat University, 23119 Elazig, Turkey; ozercanih@firat.edu.tr; 4 Department of Experimental Therapeutics, The University of Texas MD Anderson Cancer Center, Houston, TX 77030, USA; nkabil@mdanderson.org (N.K.); bozpolat@houstonmethodist.org (B.O.); 5 Winship Cancer Institute, Emory University, Atlanta, GA 30322, USA; omer.kucuk@emory.edu; 6 Houston Methodist Research Institute and Methodist Neal Cancer Center, Houston, TX 77030, USA

**Keywords:** curcumin, prevention, spontaneous leiomyoma, laying hen

## Abstract

Background: Leiomyoma (LM) is the most commonly identified tumor in the genital tract, occurring in 70–80% of women. The only treatment option is surgery, which significantly influences healthcare costs and negatively influences women’s survival and reproductive capacity. Therefore, identifying safe and effective chemopreventive and treatment modalities is needed. Methods: We investigated the effects of 12 months of daily curcumin (0, 25.8, and 53 mg/kg) diet on the incidence and growth of spontaneously developing LM tumors in a galline (hen) model. Results: LM tumors were detected in 58.9% (53/90) of the control hens as spontaneous occurrences, while they were observed in 37.7% (34/90) and 24.5% (22/90) of hens treated with daily doses of 25.8 mg or 53.0 mg, respectively, over 12 months. This reduced LM development by 35% and 58.5%, respectively (*p* = 0.004). We also observed a dose-dependent inhibition of LM-tumor growth and NF-κB, mTOR, p70S6K1, and 4E-BP1 signaling while inducing Nrf2/HO1 pathway induction LM tumors collected from hens fed with curcumin (*p* < 0.05). Curcumin intake notably reduced levels of TGF-β1, α-SMA, and collagen type 1, with dose-dependent effects (*p* < 0.001). Conclusions: The findings suggest that daily curcumin consumption significantly reduces the incidence of naturally occurring LMs and suppresses tumor growth. This indicates that regular curcumin intake may be an effective preventive measure against LMs.

## 1. Introduction

Uterine leiomyomas (LM) (also known as fibroids or myomas) are benign and the most commonly diagnosed gynecologic tumors in women in the US [1]. The lifespan occurrence of LM is exceptionally high, and by the age of 50, they are detected in about 80% of African American women and 70% of Caucasian women [1]. LM is the most common cause of gynecological surgeries and hysterectomies (200,000 cases/year) in the USA [2]. Surgery is the only definitive approach associated with surgical-related side complications, causing a significant impact on women’s lives and healthcare costs. Therefore, identifying highly effective chemopreventive strategies is urgently needed to reduce the incidence and significantly improve the quality of women’s lives and reproductive ability, potentially lowering economic burden and healthcare-related costs.

The development and progression of LM are highly complex and multifactorial, and genetic factors and hormonal abnormalities are considered essential factors contributing to the pathogenesis [3]. Previous studies have demonstrated that signaling pathways, including inflammatory pathways such as interleukin-1α (IL-1α), IL-1β, and PGE2, growth factor (EGF), transforming growth factor (TGF)-β, vascular endothelial growth factor (VEGF), and estrogen may be underlying factors in the pathogenesis of LMs [4,5,6,7]. TGF-β is vital in uterine fibroid pathophysiology, mediating cellular migration, fibroid growth, and metabolism. Upregulation of TGF-β signaling in uterine fibroids results in excessive extracellular matrix production and deposition [8]. TGF-β induces a variation of tissue fibroblasts into myofibroblasts, α-smooth muscle actin (α-SMA) expression, and collagen synthesis to form an extracellular matrix. With TGF-β activation, Smad3/4 assembles and combines with TGF-β to form a complex that migrates from the cytoplasm to the nucleus, leading to the transcription of fibrosis-related genes such as α-SMA and collagen type 1.

Curcumin, a polyphenolic compound derived from turmeric, is widely recognized for its potent antioxidant and anti-inflammatory properties [9,10]. Preclinical research has demonstrated its chemopreventive effects against various cancer types, including colon, pancreatic, and breast cancers [11,12,13,14,15,16,17,18]. Additionally, populations with diets rich in curcumin show significantly lower rates of ovarian cancer [19,20]. However, its effect on the development of LM remains incompletely understood. Promising outcomes in cancer prevention and treatment have been observed in animal models for breast cancer, esophageal cancer, hepatocellular carcinoma, and colon cancer [21,22,23,24,25,26]. Curcumin’s biological activity is mediated through mechanisms such as suppression of survival pathways, enhancement of apoptotic processes, anti-inflammatory actions, and neutralization of reactive oxygen species (ROS) [10]. Despite these benefits, curcumin faces significant criticism due to its poor pharmacokinetic and pharmacodynamic properties. Classified as both a PAINS (pan-assay interference compound) and IMPS (invalid metabolic panacea) compound, curcumin is characterized by instability, lack of specificity, and repeated failure in double-blinded, placebo-controlled clinical trials [27]. Its rapid degradation, low bioavailability, and inconsistent biological activity complicate its use, while its broad reactivity and tendency to aggregate in assay conditions cast doubt on its reported efficacy. These challenges highlight the need for rigorous experimental designs to thoroughly validate curcumin’s mechanisms of action and therapeutic potential [26].

Nonetheless, advancements in delivery systems and formulations are paving the way for the overcoming of these challenges. Innovative approaches, such as nanoparticle-based systems, micelles, and chemical modifications, have been developed to enhance curcumin’s solubility, stability, and absorption [26,28,29,30]. Combining curcumin with piperine, a natural alkaloid derived from black pepper, has proven to be a particularly effective strategy. Piperine inhibits hepatic and intestinal glucuronidation, a primary metabolic pathway for curcumin, significantly enhancing its systemic availability [31,32]. Research confirms that piperine co-administration can dramatically boost curcumin’s bioavailability, making it one of the most effective solutions for its pharmacokinetic limitations [33]. Specialized formulations such as CURCUWIN^®^ have also been developed to optimize curcumin delivery. By incorporating a water-attracting carrier, cellulosic derivatives, and natural antioxidants, CURCUWIN^®^ significantly enhances the bioavailability of curcuminoids. Studies have shown that CURCUWIN^®^ achieves curcumin blood levels 45.9 times higher than unformulated curcumin, 34.9 times higher than turmeric volatile oil formulations, and 5.8 times higher than curcumin phytosome formulations [34].

*Gallus gallus domesticus* (laying hens) are the only animal species spontaneously developing LMs, occurring in 35–70% of hens [35]. Thus, this hen model is a critical animal model for studying the chemoprevention of LMs and ovarian cancer [35,36]. Moreover, the hen model of LM shares similar histological and morphological features frequently occurring in epithelial subtypes of human tumors [35,37,38]. Although the causes of fibroid and ovarian cancer development are not definitive as previously demonstrated and published, galline models spontaneously develop various tumors, including fibroids and ovarian cancer, that closely mimic the molecular features of human tumors, including CA-125 expression and mutation frequency [39]. Earlier research confirmed that the aging hen (Gallus gallus domesticus) is an essential animal model for uterine leiomyoma, making it a valuable supplementary model for investigating the pathophysiology of human uterine leiomyomas [35]. In this research, proliferation tests employing the tritiated thymidine conjugate indicate that these cells reacted to the antifibrotic drug halofuginone treatment just as responsively as human cells did [35].

Recent molecular analyses of galline ovarian cancer have revealed naturally occurring genetic mutations, such as K-Ras and Her2/neu [40]. Moreover, beyond CA-125, the expression levels of mesothelin, cyclooxygenase-1, selenium-binding protein 1, E-cadherin, and VEGF are comparably altered in both human and galline tumors [41]. A study conducted in vitro showcased that curcumin suppresses the proliferation of uterine leiomyoma cells by modulating the apoptotic pathway. It also curtails the synthesis of the extracellular matrix component, fibronectin. These findings suggest that curcumin could offer a new avenue for leiomyoma treatments [42]. The current study results indicate that a daily curcumin intake (CURCUWIN) for 12 months dose-dependently reduces the incidence of spontaneously developing LM and the growth of LM tumors in hens. Curcumin dose-dependently reduces the expressions of NF-κB, mTOR, p70S6K, and 4E-Bp1 and induces the Nrf2/HO1 antioxidant pathway, suggesting that curcumin can be used for chemoprevention of LMs.

The objective of this study was to evaluate the chemopreventive potential of curcumin against uterine fibroids (LMs) utilizing the Gallus gallus domesticus (laying hen) model, the sole species that spontaneously develops LMs. By analyzing the impact of prolonged curcumin supplementation on tumor incidence, growth, and molecular signaling pathways (NF-κB, mTOR, p70S6K1, 4E-BP1, Nrf2/HO1), this research aims to uncover curcumin’s mechanisms of action and assess its viability as a dietary intervention for LM prevention.

## 2. Results

### 2.1. Incidence and Size of Leiomyoma

**Figure 1:**
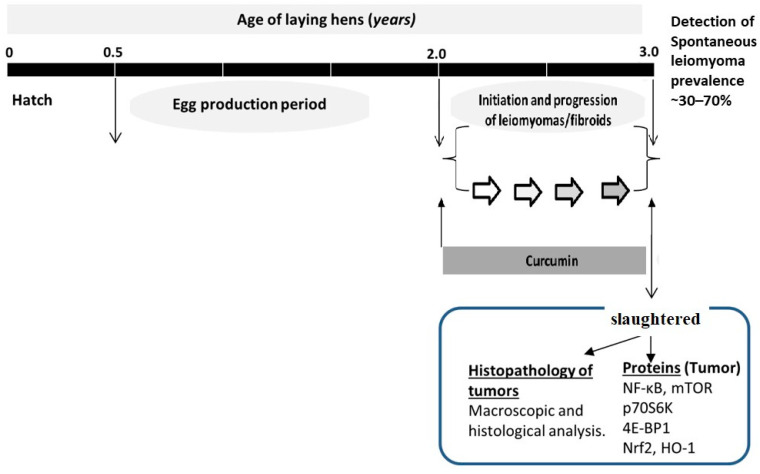
Study design in the hen model.

**Figure 2:**
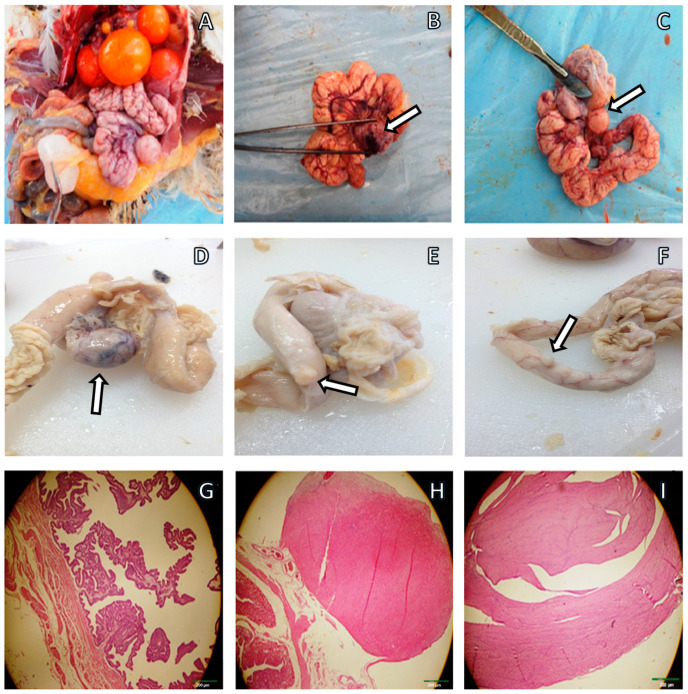
Gross morphology of oviduct LMs (**A**–**F**) in laying hens. Morphology of the normal oviduct (**G**) and histopathology of the LM tumors (**H**,**I**) in laying hens (HxE, ×40). The photographs display several nodular leiomyomas originating from the magnum’s smooth muscle layer (**A**–**F**, Nikon D100, Nikon Corporation, Tokyo, Japan).

The hens used in this study spontaneously develop LM tumors at high occurrence rates, ranging from 35% to 70%. To examine the influence of curcumin consumption on the spontaneous growth of LM incidence, hens were fed with two different doses of curcumin (0, 25.8, and 53.0 mg/day) for 12 months, and the LM prevalence was assessed after euthanasia (Figure 1). The macroscopic and microscopic appearances of LMs are shown in Figure 2A–F, G–I, respectively. In 90 hens of the control group that was not fed with curcumin, LMs spontaneously developed in 53 (58.88%), while it did so in 34 (37.77%) and 22 (24.48%) of the hens in the 25.8 and 53.0 mg/day groups (*p* < 0.004) (Table 1). Compared with the control group, we found a reduction in the overall LM incidence in these two curcumin-fed groups (35% and 58.49%, respectively). Next, we determined the effect of curcumin consumption daily on the number of tumors and tumor size. We observed a reduced number of LM tumors in each hen in the curcumin-supplemented groups compared with the control hens (3.06 ± 0.39, 1.97 ± 0.28, and 1.64 ± 0.24) (Table 1). Tumor sizes were notably smaller in the curcumin-fed groups compared with the control group (4.35 ± 0.32, 3.43 ± 0.33, 2.61 ± 0.24 in the 0, 25.8, and 53.0 mg/day groups, respectively; *p* = 0.008) (Table 1).

### 2.2. Oviduct Malondialdehyde Levels

**Figure 3:**
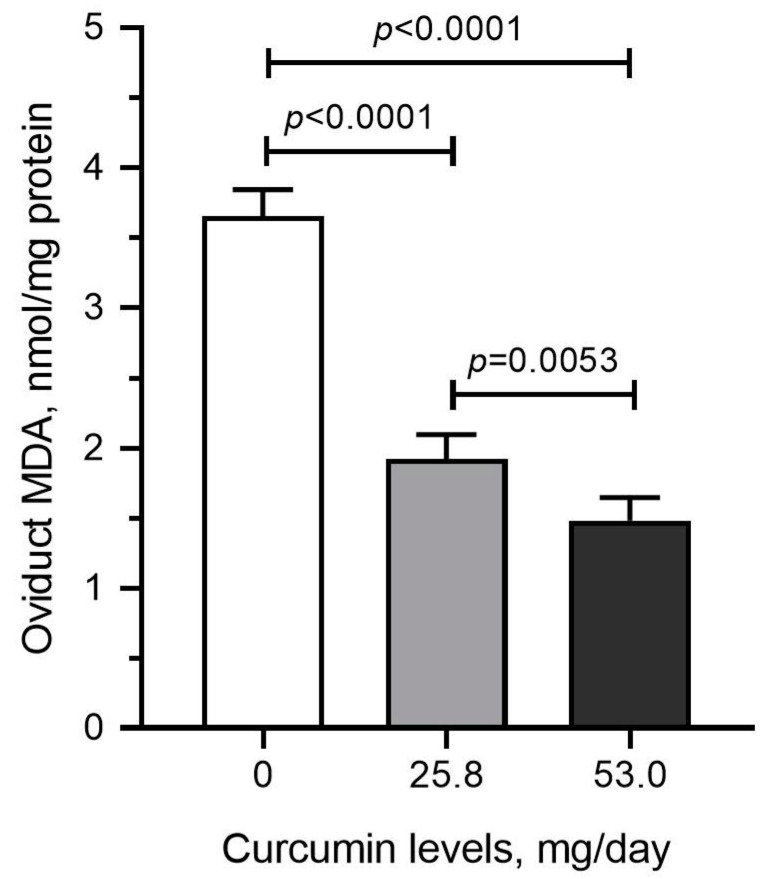
Dietary curcumin reduces MDA levels in tissues. ANOVA and Tukey’s post hoc tests compared the results in control and treatment groups (*p* < 0.05).

To determine the effect of curcumin supplementation on oxidative stress, we analyzed MDA levels, a biomarker for lipid peroxidation, in oviduct tissue samples. We found that the levels of MDA were significantly and dose-dependently reduced in the oviduct tissues collected from curcumin-fed animals compared with those collected from the control hens (*p* < 0.001) (Figure 3).

### 2.3. Oviduct NF-κB, mTOR, p70S6K, 4E-Bp1, Nrf2, and HO-1 Levels

**Figure 4:**
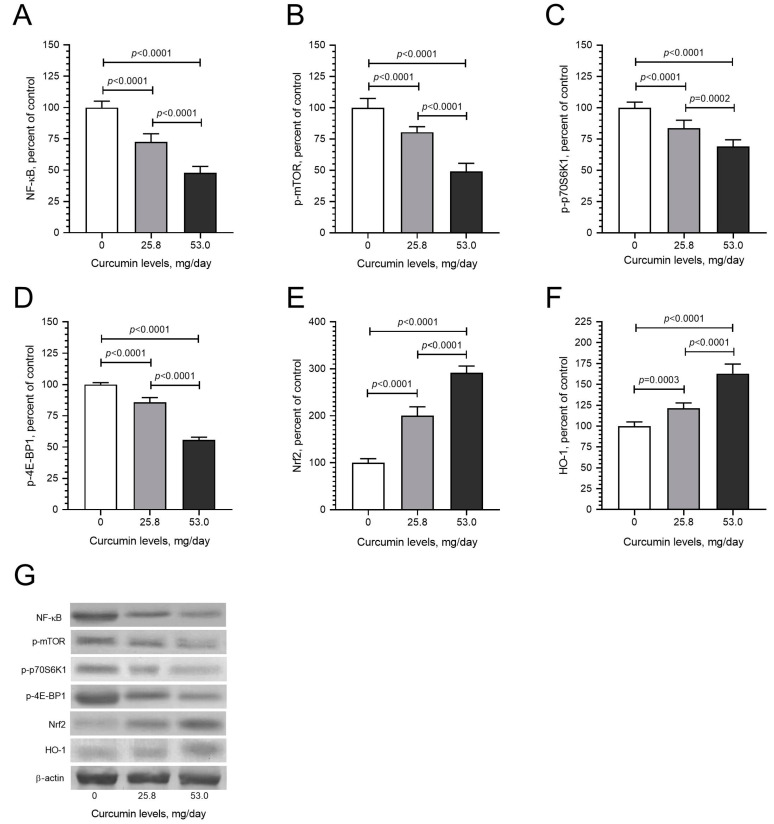
Effects of daily dietary curcumin intake (0, 25.8, and 53 mg/kg) on NF-κB (**A**), p-mTOR (**B**), p-p70S6K1 (**C**), p-4E-BP1 (**D**), Nrf2 (**E**), HO-1 (**F**) levels and representative bands (**G**) in the tissues detected by Western blot analysis (full immunoblot bands are shown in Supplementary Figure S1). Values represent the means of three different analyses. Data are the percent of the control. ANOVA and Tukey’s post hoc tests compared the different treatment groups (*p* < 0.05).

**Figure 5:**
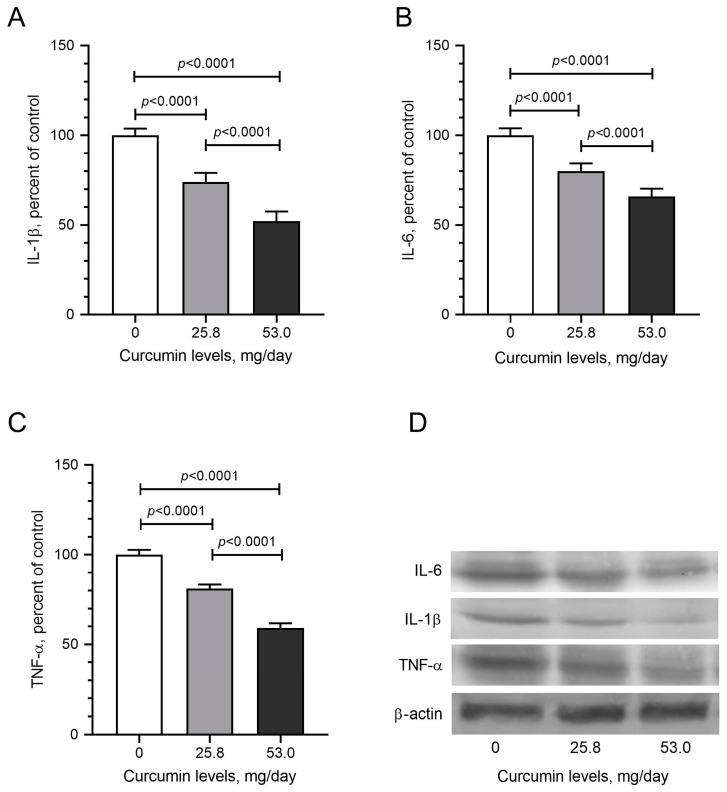
Effects of daily dietary curcumin intake (0, 25.8, and 53 mg/kg) on IL-1β (**A**), IL-6 (**B**), and TNF-α (**C**) levels and representative bands (**D**) in the tissues detected by Western blot analysis (full immunoblot bands are shown in Supplementary Figure S2). Values represent the means of three different analyses. Data are the percent of the control. ANOVA and Tukey’s post hoc tests compared the other treatment groups (*p* < 0.05).

**Figure 6:**
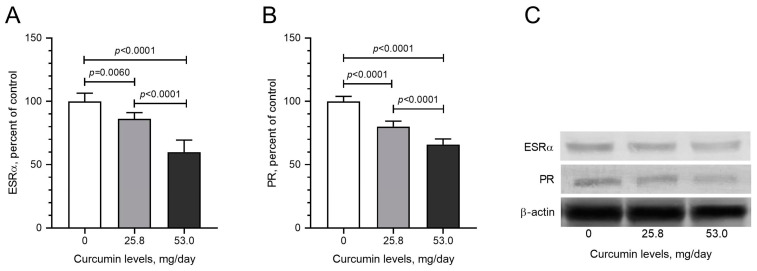
Effects of daily dietary curcumin intake (0, 25.8, and 53 mg/kg) on ESRα (**A**) and PR (**B**) levels and representative bands (**C**) in the tissues detected by Western blot analysis (full immunoblot bands are shown in Supplementary Figure S3). Values represent the means of three different analyses. Data are the percent of the control. ANOVA and Tukey’s post hoc tests were used to compare the other treatment groups (*p* < 0.05).

**Figure 7:**
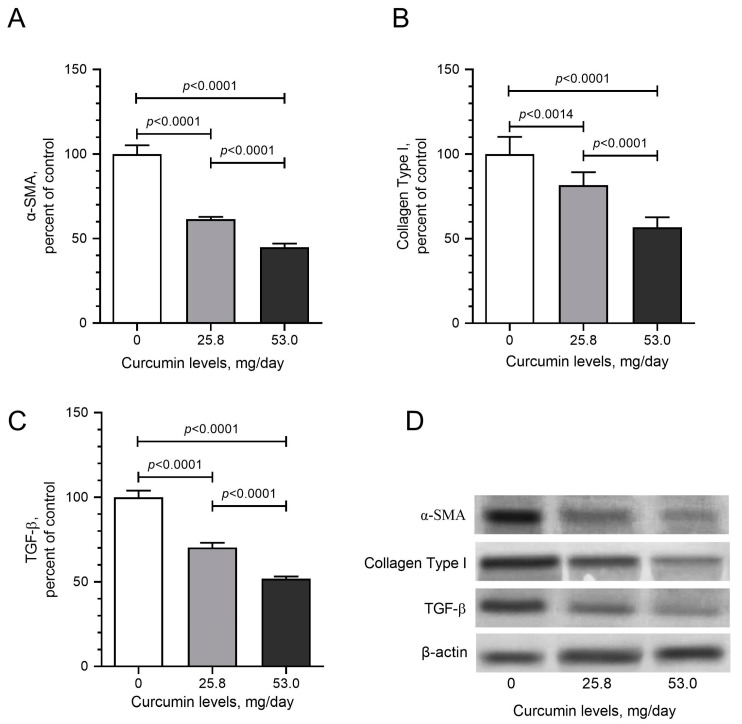
Effects of daily dietary curcumin intake (0, 25.8, and 53 mg/kg) on α-SMA (**A**), collagen type 1 (**B**) and TGF-β (**C**) levels, and representative bands (**D**) in the tissues detected by Western blot analysis. Values represent the means of three different analyses. Data are the percent of the control. ANOVA and Tukey’s post hoc tests compared the other treatment groups (*p* < 0.05).

To determine the molecular mechanism of curcumin-induced effects in LM tumors, tissue samples from the control and curcumin-fed groups were examined to express mediators of critical signaling pathways. Due to the critical role of NF-κB in cancer signaling and the well-established role of curcumin in NF-κB modulation, we first evaluated the expression of the inflammation mediator NF-κB and found that daily curcumin intake led to the inhibition of NF-κB levels by about 42% detected by Western blot analysis (Figure 4A; *p* < 0.05). Moreover, curcumin intake significantly decreased mTOR, p70S6K, and 4E-Bp1 levels compared with control hens (Figure 4B–D; *p* < 0.05). The Nrf2 antioxidant mechanism plays a vital role in cancer prevention. Nrf2 induces antioxidant proteins, including HO-1. Increased HO-1 reduces the formation of oxidants. In the tumor samples obtained from curcumin-fed animals, we found dose-dependent induction in the levels of Nrf2 (Figure 4E) and HO-1 (Figure 4F). Western blot analysis detected the effects of daily dietary curcumin intake (0, 25.8, and 53 mg/kg) on pro-inflammatory cytokines, including IL-1β, IL-6, and TNF-α levels in the tissues. The results indicate that curcumin intake dose-dependently reduced levels of IL-1β (Figure 5A), IL-6 (Figure 5B), and TNF-α (Figure 5C). Furthermore, daily dietary curcumin intake also reduced ESRα (Figure 6A) and PR (Figure 6B) levels in the tissues detected. Western blot analysis of TGF-β1, α-SMA, and collagen type 1 shows that daily curcumin intake led to a significant decrease in a dose-dependent manner in the expression of these proteins compared with the control group (*p* < 0.001, Figure 7). Representative Western blot bands for all proteins are shown in Figure 4G, Figure 5D and Figure 6C, respectively.

## 3. Discussion

This research demonstrates that daily consumption of curcumin at doses of 25.8 mg/day or 53.0 mg/day over approximately 12 months significantly reduces the incidence of LM by 35% and 58%, respectively. Curcumin also decreases tumor growth and the total number of tumors compared with the control group. Notably, this study is the first to show curcumin’s ability to suppress the spontaneous development of LM tumors.

Previous studies have indicated curcumin’s impact on LM cell proliferation [43], and its association with reduced cancer incidence has been established in rodent models involving various carcinogens, including adenomatous polyposis and cancers of the breast, liver, esophagus, and colon. [21,25,44]. However, the direct relationship between curcumin consumption and LM development has not been confirmed until now. In this study, we show for the first time that daily curcumin consumption diminishes the growth of spontaneous LMs. The hen model is an emerging animal model for LM development studies, growth, and progression, as this is the unique model of spontaneously developing LMs with a high prevalence rate [35]. This model is also a precious system for spontaneously developing ovarian cancer, which has similar pathological, histological, and molecular features to humans and shares common molecular alterations such as mutations of p53, BRCA, VEGF, and EGFR [39,45], indicating that hen models could be an excellent value for evaluating the chemopreventive role of dietary compounds in human gynecological tumors. In addition, we previously measured serum curcumin levels in hens fed with similar dietary doses of curcumin [40]. In that study, curcumin was undetectable in the serum of control hens, but its levels increased dose-dependently in curcumin-fed hens, reaching 0.071 and 0.116 μmol/L in the 25.8 and 53.0 mg/day groups. These findings confirm a systemic absorption of dietary curcumin, albeit at modest levels. Notably, curcumin’s biological effects are likely mediated through localized interactions and systemic modulation of key signaling pathways rather than requiring high circulating levels.

NF-κB is one of the primary mediators of inflammation, induces cell proliferation, and helps cells evade apoptosis [1,46,47]. Thus, the inhibition of NF-κB is a crucial mechanism for cancer prevention and therapy [14,47]. Curcumin-mediated chemopreventive and antitumor effects are mediated by inhibiting NF-κB signaling [10,48]. Daily curcumin consumption dose-dependently inhibits NF-κB expression in oviduct tissues removed from hens during the curcumin diet, supporting the contention that reduced NF-κB expression could be involved in curcumin-induced effects.

Curcumin leads to the upregulation of antioxidation-related proteins; for example, Nrf2 plays a critical role in antioxidant signaling and cancer prevention by protecting cells and tissues from various oxidative stresses [49]. Nrf2 has been shown to induce the expression of HO-1 by binding to the antioxidant response element [50,51,52]. Our study indicates that the expression in oviduct tissues removed from the hens during the curcumin diet led to a significant induction of NrF2 and HO1, suggesting that daily curcumin intake leads to antioxidant signaling and might be an important factor leading to preventive and therapeutic effects against LM. Our study also demonstrates that MDA, a well-established lipid peroxidation biomarker for evaluating the oxidant damage induced by free radicals, was significantly reduced with curcumin intake in tissues obtained from the hens. Curcumin has been previously reported to decrease in vivo MDA levels in animal models [53], which may also contribute to the chemopreventive effects of curcumin.

PI3K/Akt is known as one of the critical signaling pathways promoting tumor growth and the survival of cancer cells in tumors [54]. The activation of PI3K results in the phosphorylation of the lipid phosphatidylinositol-4,5-bisphosphate, commonly referred to as PIP2, on the plasma membrane. This transforms PIP2 into phosphatidylinositol-3,4,5-trisphosphate, also known as PIP3. The presence of PIP3 subsequently triggers the activation of Akt and PDK1. This, in turn, regulates the mammalian target of rapamycin (mTOR), leading to the activation of p70S6K and 4E-BP1 as well as Bcl-2 family proteins. These pathways are crucial in cell survival, cell cycle progression, and apoptosis. The PI3K/Akt/mTOR signaling has been linked to uterine leiomyoma [55]. The aberrant activation of the PI3K/Akt/mTOR pathway in humans and Eker rat animal models previously suggested its role in LM development and growth. Therefore, the inhibition of mTOR and its downstream mediators p70S6K and 4E-BP1 may be essential for curcumin-induced chemopreventive and therapeutic effects.

Inflammatory factors have been linked to carcinogenesis, and anti-inflammatory drugs are believed to play an important role in prevention. Curcumin is known to exert its anti-inflammatory effects by suppressing the many pro-inflammatory cytokines [9,10]. Our study also demonstrates that dietary curcumin intake significantly and dose-dependently reduced the expression of pro-inflammatory cytokines, including IL-1β, IL-6, and TNF-α levels in the tissues. Progesterone induced the development and proliferation of leiomyoma cells, while estradiol increased the effects of progesterone by increasing the availability of progesterone receptors [4,5,6]. The selective estrogen receptor modulator (SERM) raloxifene and the selective PR modulator (i.e., mifepristone) have inhibited fibroid growth in clinical trials. Therefore, the role of steroids is critical for leiomyoma development and maintenance [4,5,6]. In the current study, we found that daily dietary curcumin intake also reduced ESRα and PR levels, suggesting that it leads to multiple effects that are underlying its chemopreventive effects.

Previous studies have shown that TGF-β elevates the production of numerous extracellular matrix proteins linked to tissue scarring [56]. Moore et al. [57] demonstrated that fibroblasts originating from uterine leiomyomas promote the proliferation of uterine leiomyoma cells and the production of collagen type 1. They also observed an activation of TGF-β-receptor signaling in these co-cultures. A previous study reported that α-SMA-positive and desmin-negative cells as well as a large amount of collagen in leiomyoma tissue indicate the presence of myofibroblasts and their role in the ECM deposition [58]. In the current study, we observed decreased levels of TGF-β1, α-SMA, and collagen type 1 in the tissues of hens fed with curcumin. The curcumin-induced suppression of TGF-β1 activation, which is induced by inflammatory cytokines, may be one of the critical mechanisms of chemopreventive and therapeutic effects as TGF-β signaling has been reported to function as a tumor suppressor and to play a critical role in preventing carcinogenesis in normal epithelial cells by inducing cell cycle arrest, apoptosis, and the prevention of cell immortalization [59].

The administration of curcumin to humans is well tolerated at up to 8 g/day, with no side effects [60]. In our study, the high dose of curcumin was 53.0 mg/day, corresponding to 1.4 g/day in humans, suggesting that it can be easily achievable [61]. Thus, future studies should consider these doses to evaluate the chemopreventive role of curcumin for LMs in clinical trials.

While these findings underscore the potential of curcumin as a dietary intervention for LMs, limitations such as the use of a single animal model and the absence of detailed pharmacokinetic data necessitate further research. Additionally, a key limitation of this study is the lack of a control group receiving the CURCUWIN formulation with curcumin replaced by an inert compound. This would have allowed us to distinguish the effects of curcumin from those of other bioactive components in the CURCUWIN formulation, such as water-attracting carriers, cellulosic derivatives, and natural antioxidants. Including such a control group in future studies would help delineate the specific contributions of curcumin and its formulation to the observed outcomes. Additionally, integrating advanced technologies, including omics and PROTAC probes [62], could provide deeper insights into curcumin’s molecular targets and mechanisms. Future clinical studies evaluating curcumin’s efficacy at achievable human doses will be essential to validate its role in LM management and explore its broader implications for gynecological and other tumor types.

## 4. Materials and Methods

### 4.1. Hens and Diets

A total of 270 Brown laying hens (104 weeks old; White Leghorn W96 strain) were kept in cages under regulated temperature and humidity conditions. This research took place at the Veterinary Control and Research Institute in Elazig, Turkey. All experimental procedures involving animals were permitted by the Animal Experimentation Ethics Committee of the same institute (Elazig, Turkey; 013/1-1). All implemented procedures were conducted in strict compliance with the relevant laws, the Animal Welfare Act, the Public Health Services Policy, and the standards set by the Institute’s Institutional Animal Care and Use Committee.

Hens in cages were fed 1 of 3 diets (*n* = 90); a standard diet (0) and a standard diet (Supplementary Table S1) were added with either 200 mg of curcumin per kilogram of diet (200) or a basal diet added with either 400 mg of curcumin per kilogram of diet (400) for 12 months. The curcumin doses used in this study were determined based on our previous study [40]. Curcumin (CURCUWIN) was provided by OmniActive Health Technologies (Bridgewater, NJ, USA). Hens in the curcumin groups received a standard diet that provided 25.8 mg and 53.0 mg of curcumin daily. The hens consumed approximately 130 g per day of feed with or without curcumin. The diet composition is given in Supplementary Table S1. The animal had free access to water and feed ad libitum. The hens were kept on a daily artificial photoperiod (16L:8D) of 16 h using an automated lighting control system. Diets were batch-prepared weekly and stored in dark plastic containers at 4 °C to shield them from photo-oxidation. The food containers holding the diet were kept shielded from light and replaced weekly.

### 4.2. Sample Collection

At the end of the study, blood samples were collected and centrifuged for 10 min. Subsequently, the hens were slaughtered. The presence or absence of tumors in the oviduct and their sizes were documented. Serum and tissue specimens were preserved at −80 °C for future use. Tissue samples underwent fixation in 10% neutral buffered formalin before being embedded in paraffin. The samples were then evaluated for morphological and histological variations, and tissue types of tumors were identified through hematoxylin and eosin staining of the tissue slices [63,64]. Each 6 µm thick tissue section was sliced from each paraffin block. An expert histopathologist, unaware of the study groupings, inspected these sections under a light microscope to identify any pathological characteristics of LM.

### 4.3. Malondialdehyde Concentrations

The level of Malondialdehyde (MDA), an indicator of oxidative stress, in the oviduct was determined using HPLC equipped with an SPD-10A UV-detector (Shimadzu, Kyoto, Japon) following a method previously outlined [63]. For the analysis, tissue samples weighing 300 mg were homogenized and centrifuged, and the resulting supernatants (with an injection volume of 20 µL) were introduced into the HPLC system. The employed mobile phase consisted of 30 mM KH2PO4 and methanol (82.5 + 17.5, v/v%, pH 3.6), a 1.2 mL/minute flow rate, and a detection at 250 nm.

### 4.4. Immunoblot Analysis

NF-κB, p-mTOR, p70S6K, 4E-Bp1, Nrf2, HO-1, IL-1β, IL-6, TNF-α, ESR-α, PR, TGF-β1, α-SMA, and collagen type 1 were extracted from oviduct tumors for Western blot evaluation as defined earlier [65]. Samples were homogenized and centrifuged; the supernatant was transferred into fresh tubes for analysis. An amount of 20 µg of protein was subjected to 10% SDS-PAGE and then transferred to nitrocellulose membranes. Nitrocellulose blots were blocked with 1% bovine serum albumin. NF-κB (bs-0465R), p-mTOR (bs-1992R), p-P70S6K (bs-6370R), p-4E-BP1 (bs-3018R), Nrf2 (bs-1074R), HO-1 (bs-2075R), IL-1β (bs-0812R), IL-6 (bs-0782R), TNF-α (bs-0078R), ESR-α (bs-2098R) (Bioss, MA, USA, for all), PR (Abcam, ab191138), TGF-β1 α-SMA (Invitrogen, PA5-85919) and collagen type 1 (Bioss, bs-10423R) were diluted at 1:1000 in the buffer containing 0.05% Tween-20. Blots were cut prior to hybridization with antibodies during blotting (Figures S1–S4).

The nitrocellulose membrane underwent overnight incubation at 4 °C with antibodies. To ensure consistent protein loading, an anti-β-actin antibody was utilized. Densitometric analysis was performed to assess protein levels using the ImageJ software, 1.49 a program developed by the National Institutes of Health.

### 4.5. Statistical Analysis 

The required sample size was calculated using the G*Power software (Version 3.1.9.2), setting an alpha error at 0.05 and aiming for 85% statistical power. In the current study, the Shapiro–Wilk test was utilized to ensure that data adhered to the normality requirements of parametric tests, while the “Levene” test checked for variance homogeneity. The χ^2^ test was employed to discern statistical variances between the control and treatment groups regarding tumor occurrences and their sizes. All other data underwent analysis using the General Linear Model (GLM) in the SAS software (2002; SAS Institute Inc., version 9.0, Cary, NC, USA). If the F statistic in the analysis of variance was significant (*p* ≤ 0.05), the least squares mean procedure was utilized to differentiate means that were significantly distinct (*p* < 0.05). To ascertain the impact of curcumin, both linear and quadratic polynomial contrasts were conducted on the responses.

## 5. Conclusions

In conclusion, this study provides strong evidence of curcumin’s chemopreventive and therapeutic potential against leiomyoma. Daily curcumin intake significantly reduced tumor incidence, growth, and count, with effects mediated through multiple mechanisms, including the inhibition of NF-κB signaling, the activation of antioxidant pathways (Nrf2/HO-1), the suppression of PI3K/Akt/mTOR signaling, the reduction in inflammatory cytokines, and the modulation of steroid receptors. Curcumin also decreased oxidative damage and extracellular matrix deposition, underscoring its multifaceted role in LM prevention and therapy.

## Figures and Tables

**Table 1:** Effect of curcumin intake on the growth of fibroid tumors in laying hens.

Item	Dietary Curcumin Intake, mg/Day			*p*
	0	25.8	53	
Incidence (%) ^1^	(53/90) 58.88 ^a^	(34/90) 37.77 ^b^	(22/90) 24.48 ^b^	0.001 X^2^ = 22.55
LM tumor number/hen	3.06 ± 0.39 ^a^	1.97 ± 0.28 ^ab^	1.64 ± 0.24 ^b^	0.02
LM size (mm)	4.35 ± 0.32 ^a^	3.43 ± 0.33 ^ab^	2.61 ± 0.24 ^b^	0.008
LM size range (mm)	1–18	1–13	1–7	-

Data are the least square means from a 12-month hen study. ^a,b^: Means in the same line without a common superscript differ significantly (*p* < 0.05). ^1^ Comparison was made by Fisher’s test.

## Data Availability

The data supporting this study’s findings are available from the corresponding author upon reasonable request.

## Supplementary Materials

The following supporting information can be downloaded at: https://www.mdpi.com/article/10.3390/ph17121732/s1, Table S1. Composition of the basal diet; Figure S1. Full immunoblots related to Figure 4 [NF-kB (A), p-mTOR (B), p-p70S6K1 (C), p-4E-BP1 (D), Nrf2 (E), HO-1 (F), and β-actin (G)]; Figure S2. Full immunoblots related to Figure X [IL-1β (A), IL-6 (B), TNF-α (C), and β-actin (D)]; Figure S3. Full immunoblots related to Figure X [ESRα (A), PR (B), and β-actin (C)]; Figure S4. Full immunoblots related to Figure X [α-SMA (A), Collagen Type I (B), TGF-β (C), and β-actin (D)].
