# Supplementary material for: The Role of Curcumin in Preventing Naturally Occurring Leiomyoma in the Galline Model
Source: Pharmaceuticals (Basel). 2024 Dec 21;17(12):1732. doi: 10.3390/ph17121732 (PMC11677480; doi:10.3390/ph17121732)
Supplement: Supplementary file 1 [file pharmaceuticals-17-01732-s001.zip › pharmaceuticals-3340811-supplementary.pdf]

Table S1: Composition of the basal diet

Diet Ingredients	g/kg (of diet)
Corn	630.0
Soybean meal	245.8
Tallow	22.7
Limestone	89.0
Dicalcium phosphate	2.5
Mineral premix*	3.0
Vitamin premix*	3.0
Sodium chloride	2.0
Sodium bicarbonate	2.0
Chemical analyses, dry matter basis, %	
Crude protein	17.18
Crude fat	4.78
Crude fiber	3.32
Crude ash	11.58
Calcium	3.89
Phosphorus	0.32
Calculated compositions	
Methionine	0.40
Lysine	0.82
Metabolizable energy, kcal/kg	2800

*The diet was supplied per kilogram of diet: retinyl acetate, 41.28 mg; cholecalciferol, 60 µg; dl-α-tocopheryl acetate, 30 mg; menadione sodium bisulfite, 2,5 mg; thiamine-hydrochloride, 3 mg; riboflavin, 7 mg; niacin, 40 mg; d-pantothenic acid, 8 mg; pyridoxine hydrochloride, 4 mg; vitamin B_{12}, 0,015 mg; vitamin C, 50 mg; folic acid, 1 mg; D-biotin, 0,045 mg; choline chloride, 125 mg; Mn ($MnSO_4$-H_2O), 80 mg; Fe ($FeSO_4$-$7H_2O$), 30 mg; Zn (ZnO), 60 mg; Cu ($CuSO_4$-$5H_2O$), 5 mg; Co ($CoCl_2$-$6H_2O$), 0,1 mg; I as KI, 0,4 mg; Se (Na_2SeO_3), 0,15 mg.

(A) NF-κB

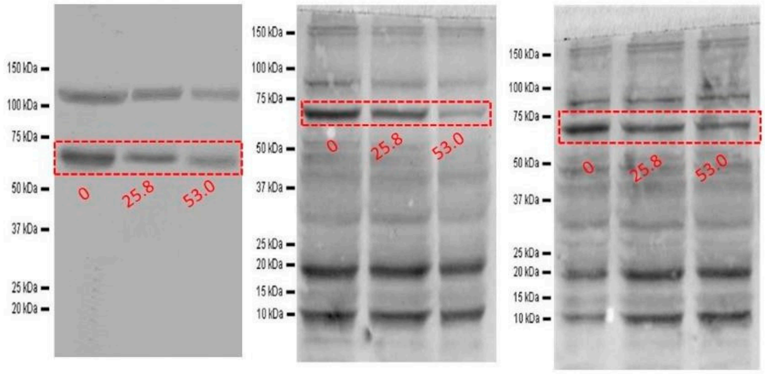

(B) p-mTOR

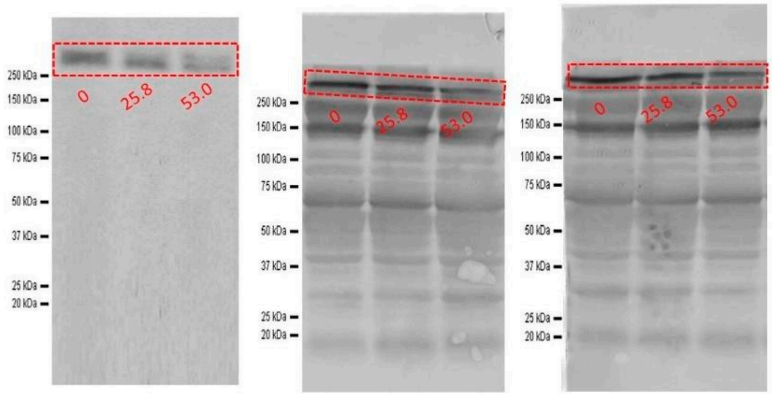

(C) p-p70S6K1

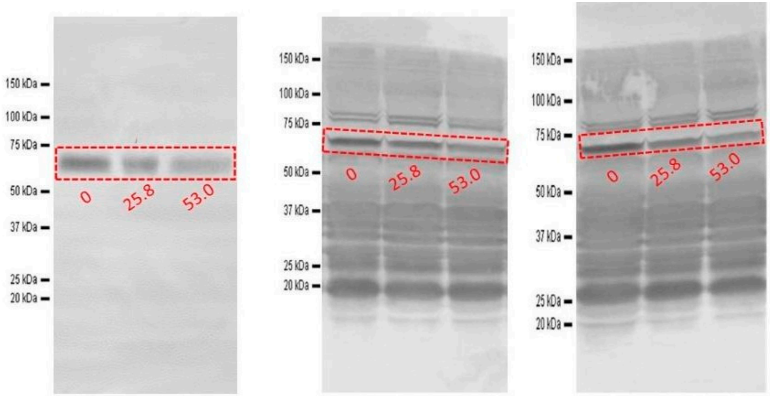

(D) p-P-4E-BP1

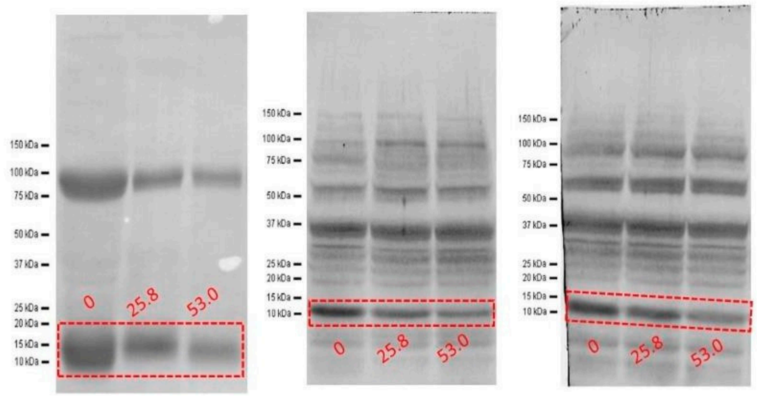

(E) Nrf2

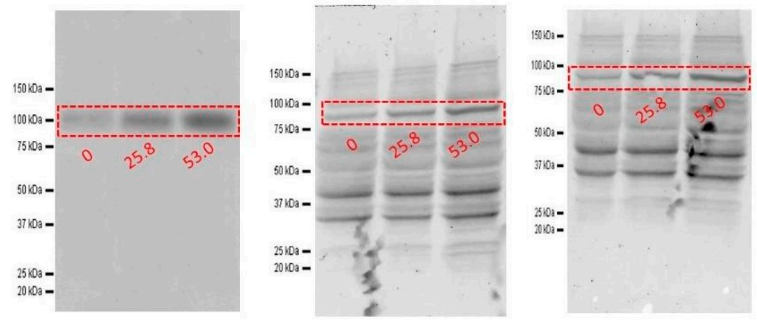

(F) HO-1

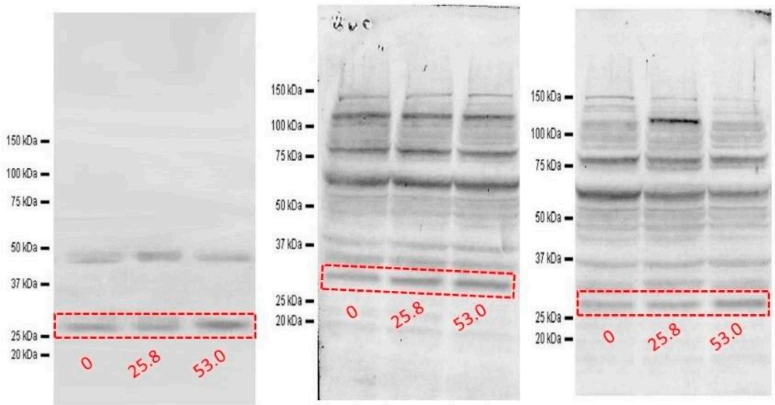

(G) β-actin

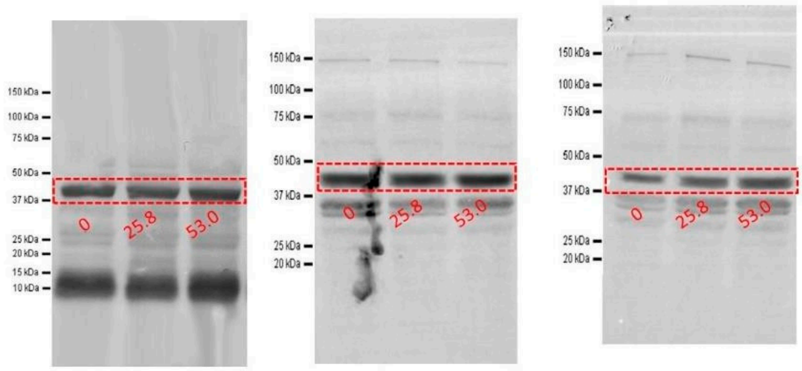

Figure S1. Full immunoblots related to Figure 4 [NF-kB (A), p-mTOR (B), p-p70S6K1 (C), p-4E-BP1 (D), Nrf2 (E), HO-1(F), and β-actin (G)]. Each immunoblot is a representative of three independent experiments. MW (in kDa) are indicated. Actin expression was used to ensure equal protein loading.

(A) IL-1β

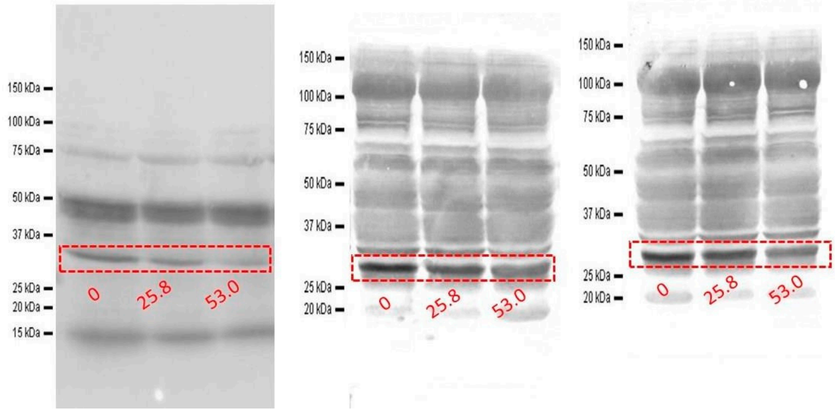

(B) IL-6

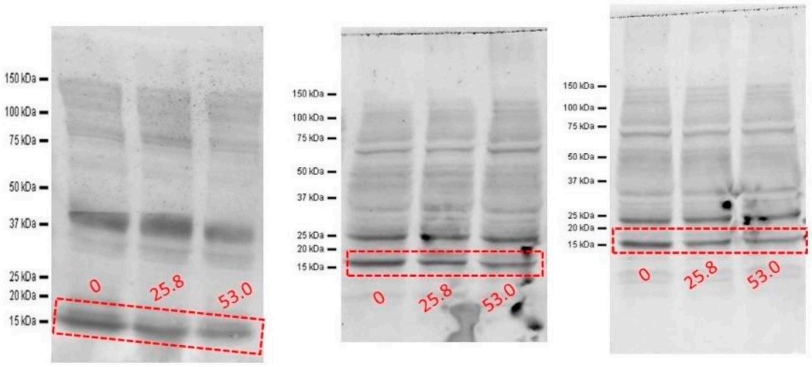

(C) TNF-α

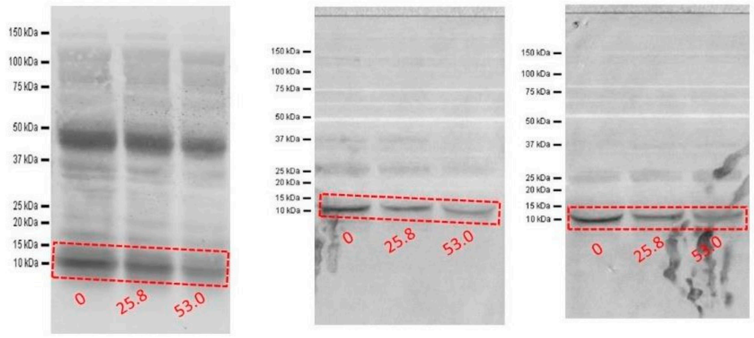

(D) β-actin

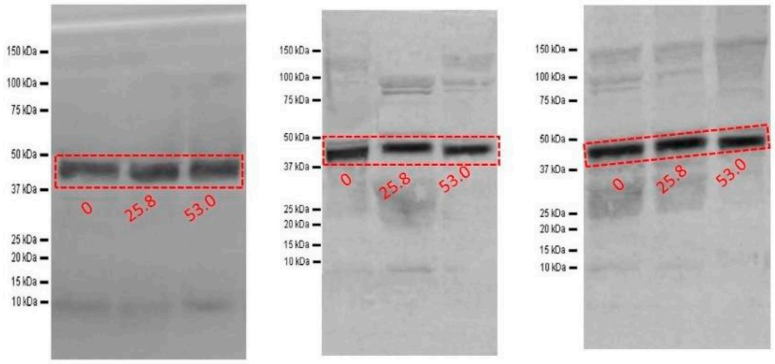

Figure S2. Full immunoblots related to Figure X [IL-1β (A), IL-6 (B), TNF-α (C), and β-actin (D)]. Each immunoblot is a representative of three independent experiments. MW (in kDa) are indicated. Actin expression was used to ensure equal protein loading.

(A) ESRα

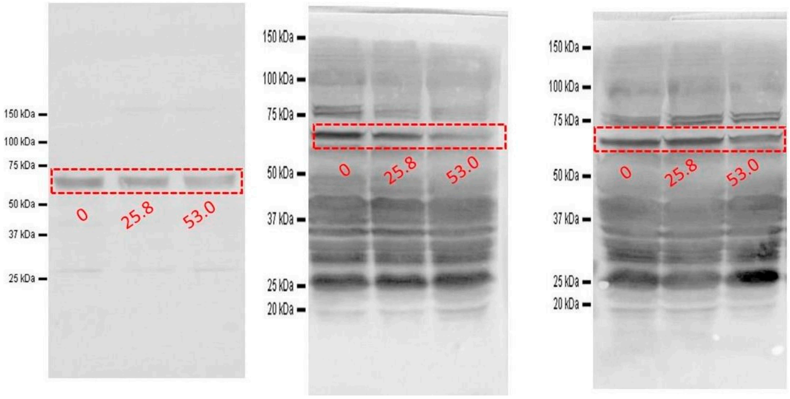

(B) PR

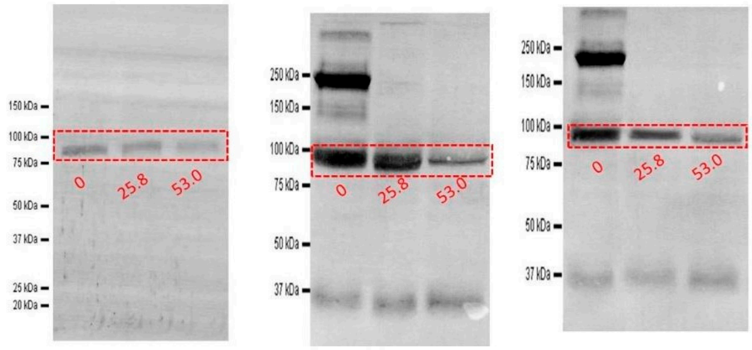

(C) β-actin

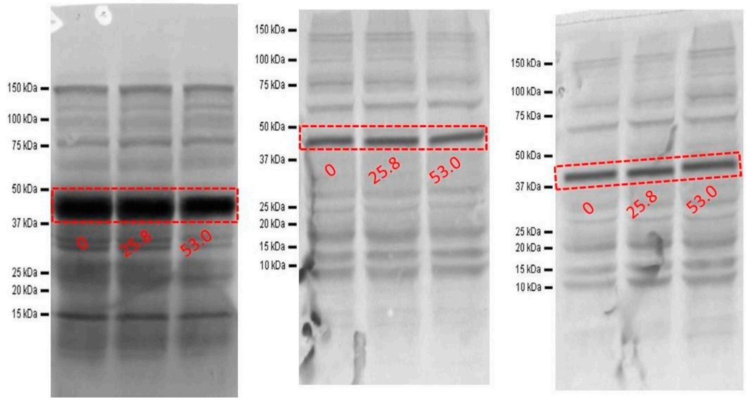

Figure S3. Full immunoblots related to Figure X [ESRα (A), PR (B), and β-actin (C)]. Each immunoblot is a representative of three independent experiments. MW (in kDa) are indicated. Actin expression was used to ensure equal protein loading.

(A) α-SMA

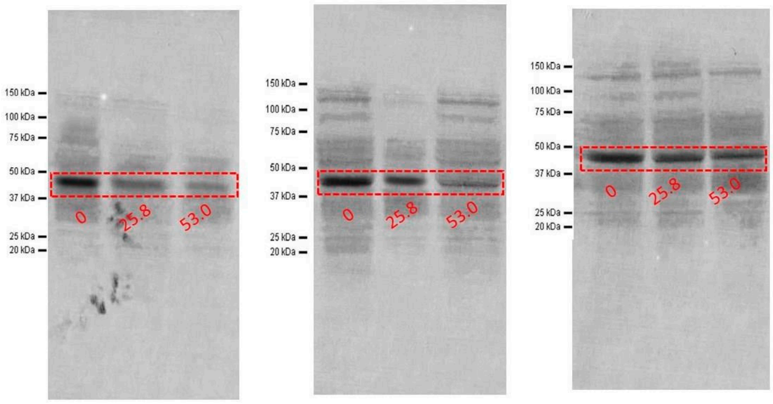

(B) Collagen Type I

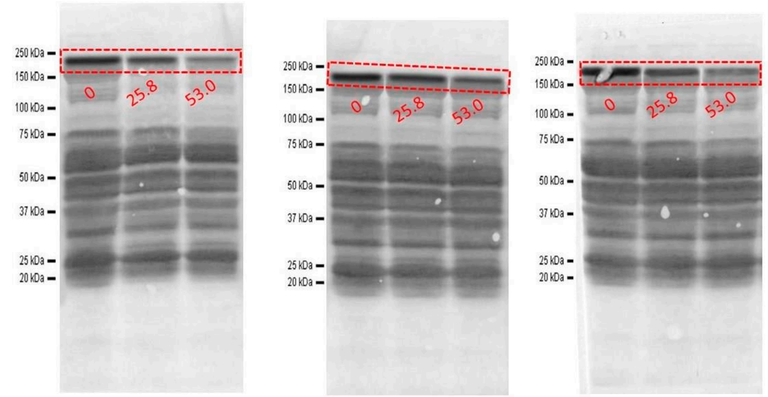

(C) TGF-β

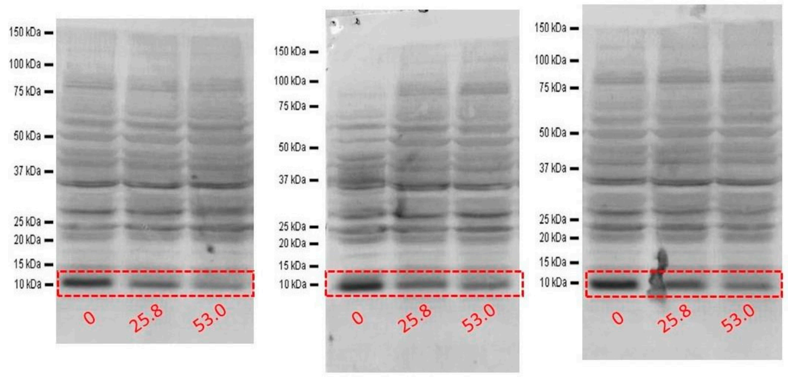

(D) β-actin

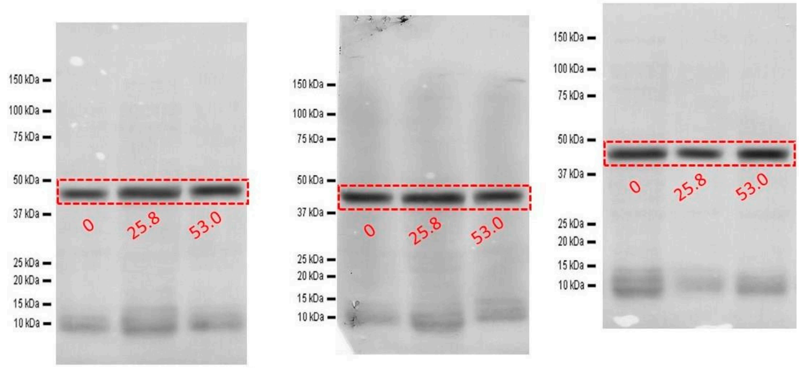

Figure S4. Full immunoblots related to Figure X [α-SMA (A), Collagen Type I (B), TGF-β (C), and β-actin (D)]. Each immunoblot is a representative of three independent experiments. MW (in kDa) are indicated. Actin expression was used to ensure equal protein loading.
